# In Situ 3D Monitoring of Geometric Signatures in the Powder-Bed-Fusion Additive Manufacturing Process via Vision Sensing Methods

**DOI:** 10.3390/s18041180

**Published:** 2018-04-12

**Authors:** Zhongwei Li, Xingjian Liu, Shifeng Wen, Piyao He, Kai Zhong, Qingsong Wei, Yusheng Shi, Sheng Liu

**Affiliations:** 1 State Key Laboratory of Material Processing and Die & Mould Technology, Huazhong University of Science and Technology, Wuhan 430074, China; zwli@hust.edu.cn (Z.L.); roya_wen@hust.edu.cn (S.W.); piyaohe@hust.edu.cn (P.H.); wqs_xn@163.com (Q.W.); shiyusheng@hust.edu.cn (Y.S.); 2 The Institute of Technological Sciences, Wuhan University, Wuhan 430072, China; 3 School of Power and Mechanical Engineering, Wuhan University, Wuhan 430072, China

**Keywords:** in situ monitoring, additive manufacturing, surface topography, contour detection, quality inspection

## Abstract

Lack of monitoring of the in situ process signatures is one of the challenges that has been restricting the improvement of Powder-Bed-Fusion Additive Manufacturing (PBF AM). Among various process signatures, the monitoring of the geometric signatures is of high importance. This paper presents the use of vision sensing methods as a non-destructive in situ 3D measurement technique to monitor two main categories of geometric signatures: 3D surface topography and 3D contour data of the fusion area. To increase the efficiency and accuracy, an enhanced phase measuring profilometry (EPMP) is proposed to monitor the 3D surface topography of the powder bed and the fusion area reliably and rapidly. A slice model assisted contour detection method is developed to extract the contours of fusion area. The performance of the techniques is demonstrated with some selected measurements. Experimental results indicate that the proposed method can reveal irregularities caused by various defects and inspect the contour accuracy and surface quality. It holds the potential to be a powerful in situ 3D monitoring tool for manufacturing process optimization, close-loop control, and data visualization.

## 1. Introduction

Additive Manufacturing (AM) has been gaining increasing attention for industrial applications in recent years [1,2,3,4,5]. Powder-bed-fusion additive manufacturing (PBF AM), such as selective laser melting (SLM) or selective laser sintering (SLS), is one of the most popular additive manufacturing techniques [6]. As the rapid and continuous technological improvements of PBF AM, the quality, and repeatability of parts produced by the PBF AM has been improved significantly. Nevertheless, some drawbacks (such as porosity, warping, inhomogeneity, etc.) still exist in the PBF AM process, due to operating with open loop control and limited monitoring of the process. All of these factors inhibit the development of the PBF AM technology to some extent.

In situ process monitoring is a suite of tools with the high potential to address the above problems [7,8,9,10]. It allows one to control the quality and stability of the AM process during the layer-wise manufacturing. Mani et al. [7] defined the quantities that can be determined during the PBF AM process as “process signatures” including dynamic characteristics of the manufacturing process. Among these process signatures, geometric signatures [11,12,13,14,15,16,17,18,19,20,21] are one of the main concerns in in situ monitoring techniques of the PBF AM process. In those early studies, several efforts used a monocular camera to implement 2D geometric measurement and defects detection [11,12,13,14,15,16]. Moreover, some researchers also considered the categorization of the potential error sources [13,14] and the verification of the deposited powder or fusion layers [15]. The research work demonstrated that the in situ 2D geometric signatures monitoring held the viability of both process improvement and characterization of internal part geometries.

However, the stated in situ monitoring methods [11,12,13,14,15,16] mostly worked with 2D images and did not yield quantitative 3D data. Investigations on in situ monitoring of 3D geometric signatures are limited, presumably because the measurement environment is challenging and directly-applicable sensors are not available. Recently, some interferometric imaging methods were conducted for 3D geometric signatures measurement of the PBF AM process [17,18]. The measurement range of PBF AM on the order of hundred millimeters are challenging for the interferometric methods with the measurement range of several millimeters. Holzmond and Li [19] presented a quality assurance system using three-dimensional digital image correlation (3D-DIC) [22] based on a fused filament fabrication (FFF) 3D printer. However, lack of enough correlation between the stereo image pairs should be reconsidered in the PBF AM process while it is practically impossible to prepare speckles over the powder bed. Zhang et al. [20,21] proposed an in situ surface 3D topography of laser powder bed fusion using monocular fringe projection. They demonstrated that this system was suitable for measuring the height profile and surface pattern of each layer. Nevertheless, it was difficult for the system to measure accurate 3D contour data at the slice level, due to limitations of the monocular fringe projection system. Thus, it is still difficult for existing techniques to obtain strictly quantitative information for powder bed, fusion and defects area during the PBF AM process.

In this work, a non-destructive in situ 3D monitoring of geometric signatures in the PBF AM process is presented based on vision sensing methods. It aims at monitoring two main categories of geometric signatures: 3D surface topography and 3D contour data of the fusion area. The analysis of the 3D surface topography properties reveals irregularities that may be caused by different defects. Because of the complex manufacturing environment and rapid production process of the PBF AM technique, the demands concerning measurement accuracy and processing velocity are always increasing. Unlike traditional phase measuring profilometry (PMP) methods that require additional images (6–12) for phase unwrapping procedure, an enhanced phase measuring profilometry (EPMP) is proposed to measure the 3D surface topography reliably without phase unwrapping. We directly find the corresponding points from wrapped phase maps. A comprehensive constraint considering both the disparity range and the image correlation constraint is proposed to select the correct corresponding point from multiple candidates. As a result, the efficiency can be remarkably improved.

Another important geometric signature concerned in this work is the 3D contour data of each layer of fusion area. However, the contour detection is always time-consuming, and the robustness is low due to the complex manufacturing and lighting environment [23,24,25,26]. To increase the accuracy and efficiency of the contour detection process, a slice model assisted contour detection method is proposed. By using prior knowledge from the slice data of each layer, the region of interest (ROI) can be located accurately in the captured images. The numbers of iterations and un-wanted false contours can be decreased with an initial set-up for the region of interest (ROI). The manual interventions are no longer needed and the contour detection process can operate automatically. Therefore, the detection efficiency and accuracy are improved significantly. The enhanced phase measuring profilometry (EPMP) and the slice model assisted contour detection constitute the technical body of the proposed in situ 3D monitoring method for the AM process. It will contribute to the development of addictive manufacturing on many levels, such as inspecting the contour accuracy and surface quality. It also holds the potential to be a powerful tool for manufacturing process optimization, close-loop control, and data visualization.

This paper is organized as follows: Section 2 introduces the theoretical principles involved in this research, including an overview of in situ 3D monitoring of geometric signatures in the PBF AM process, the enhanced PMP based 3D topography measurement and slice model assisted contour detection method, Section 3 shows the experimental validations, Section 4 discusses the strengths and potential improvement of our proposed method, and Section 5 summarizes the paper.

## 2. Principles and Methods

In this section, we will introduce the related principles of each technology used in this work, which includes an overview of the proposed in situ 3D monitoring method, the enhanced PMP based 3D topography measurement, as well as the robust and accurate contour detection assisted by slice model.

### 2.1. Overview of In Situ 3D Monitoring of Geometric Signatures in the PBF AM Process

**Figure 1:**
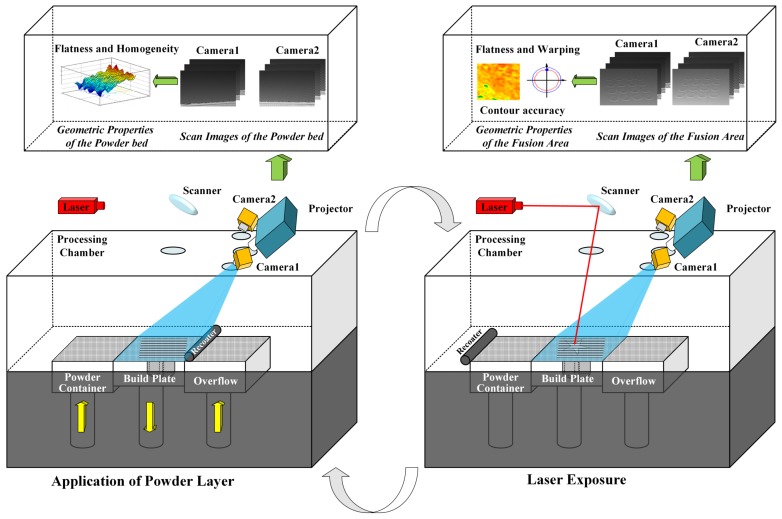
Schematic diagram for proposed in situ 3D monitoring of geometric signatures in the Powder-bed-fusion additive manufacturing (PBF AM) process.

A schematic representing a characteristic in situ 3D monitoring of geometric signatures in powder bed fusion (PBF) process is provided in Figure 1. For monitoring the 3D surface topography of powder bed, once the process of spreading powder is finished at each layer, the projector will project a series of sinusoidal fringe images onto the powder bed and the two cameras capture the fringe images synchronously. The images are then processed with an enhanced PMP method as discussed in Section 2.2. The dense 3D topography of the powder bed can be obtained and many valuable process signatures (such as the flatness, homogeneity, defects, etc.) can be directly calculated or observed.

As for monitoring the geometric properties of the fusion area, the same procedure as the powder bed measurement is applied to measure the 3D topography of the fusion area after laser exposure. In addition, a pair of stereo images is captured. The contour of the fusion area can be detected accurately and be rapidly assisted by the slice model. Based on the binocular stereoscopic vision algorithm, the 3D contour data can be obtained. The 3D topography and 3D contour data constitute the slice characteristics of each layer. The determination of the slice characteristics allows detection of super-elevated edges and other surface irregularities. After the above process, the build platform is lowered and the process is repeated layer by layer as the AM part is built from the bottom up.

### 2.2. Enhanced PMP Based 3D Topography Measurement

For a single measurement procedure, a series of sinusoidal fringe images frame with constant phase shifting is projected onto the powder bed or fused parts as shown in Figure 1. The two cameras then capture the fringe images synchronously. The distribution function of the captured images can be expressed as:

(1) $$I_{i}\left(x,y\right)=A\left(x,y\right)+B\left(x,y\right)\cos\left[\phi \left(x,y\right)+\delta_{i}\right],$$

where (x,y) is the position in the pixel coordinate system that can be omitted in the following. *A* represents the ground truth gray value of the measured object, *B* is the gray-scale modulation of the image, the principal phase value is denoted as ϕ and $\delta_{i}$ represents the known phase offset in the captured image. The sum of these means the known gray value of the recorded image. Here, *A*, *B*, and ϕ are all unknowns. Therefore, three or more images are required to calculate the principal phase value [27]. In this work, the principal phase value ϕ is solved by a least-square algorithm:

(2) $$\phi =-\arctan\left(\sum_{i=1}^{N}I_{i}\sin\left(\delta_{i}\right)/\sum_{i=1}^{N}I_{i}\cos\left(\delta_{i}\right)\right).$$

The arctangent function in Equation (2) results in a value range of [−π,π] with 2π discontinuities. Generally, a spatial or temporal phase unwrapping algorithm is required to convert the wrapped phase map to an absolute unwrapped phase map before corresponding point search [28]. This requires additional images and information. Because of the complex manufacturing environment and the rapid production process of the PBF AM technique, the demands concerning measurement accuracy and processing velocity are always increasing. Therefore, it is very important to use a minimum image number to achieve 3D surface topography.

To solve the issues, an enhanced phase measuring profilometry (EPMP) is proposed to measure the 3D surface topography reliably without capturing additional images. Instead of phase unwrapping, we directly find the corresponding points from wrapped phase maps. However, for a given pixel point in the image of camera 1, the corresponding point of it would have multiple possible positions in the image of camera 2. In order to select the correct corresponding point from multiple candidates, a comprehensive constraint considering both the disparity range and the image correlation constraint is proposed. The comprehensive constraint contains two aspects: the disparity range constraint and the image correlation constraint.

**Figure 2:**
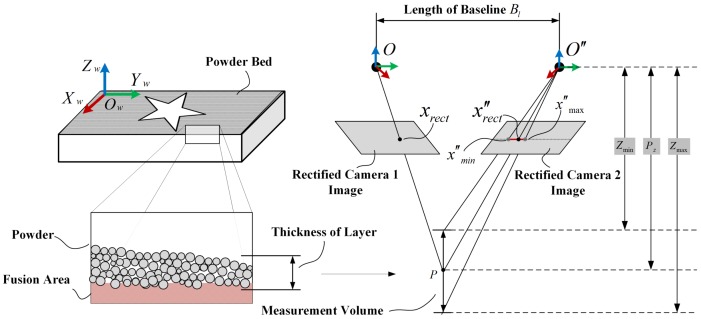
Disparity range of correspondence constrained by measurement volume of the powder bed.

The principle of disparity range constraint (DRC) is shown in Figure 2. One of the key insights, which allows DRC to decrease the number of false candidates, is that the measurement volume constrains the possible depth range within the epipolar line [29]. As shown in Figure 2, for a point $x_{rect}$ in the rectified image of camera 1, ${x^{\prime \prime }}_{rect}$ is the correct corresponding point in the rectified image of camera 2. The $Z_{min}$ and $Z_{max}$ are the minimum and maximum depths of the measurement volume. Based on the multi-view geometry, one can calculate the points ${x^{\prime \prime }}_{min}$ and ${x^{\prime \prime }}_{max}$ in the image of camera 2 corresponding to $Z_{min}$ and $Z_{max}$, respectively. ${x^{\prime \prime }}_{min}{x^{\prime \prime }}_{max}$ is constrained by the measurement volume, it can be expressed by:

(3) $$\left\{\begin{array}{c} {\left(x_{rect}-{x^{\prime \prime }}_{rect}\right)}_{\min}=F_{rect}B_{l}/Z_{\max}\Rightarrow {x^{\prime \prime }}_{min},\\ {\left(x_{rect}-{x^{\prime \prime }}_{rect}\right)}_{\max}=F_{rect}B_{l}/Z_{\min}\Rightarrow {x^{\prime \prime }}_{max}, \end{array}\right.$$

where $B_{l}$ is the baseline length, and $F_{rect}$ is the rectified focal length of the two cameras. The point ${x^{\prime \prime }}_{rect}$ must fall on the line section ${x^{\prime \prime }}_{min}{x^{\prime \prime }}_{max}$. In the meantime, measurement depth $Z_{max}-Z_{min}$ of the monitoring area is very limited that ranges from 1 to 2 mm. The search area can be narrowed down to the short line section ${x^{\prime \prime }}_{min}{x^{\prime \prime }}_{max}$ that is the red line in Figure 2. Therefore, by projecting all candidates to the rectified image plane, the ones out of disparity range will be rejected. This increases the robustness and effectiveness of the corresponding point search.

However, there are still several existing mismatched points. To solve this problem, we perform image correlation constraint to further select the final corresponding point. The image correlation [22] relies on finding the maximum of the correlation array between pixel intensity array subsets on two or more corresponding images. Therefore, it is rational that the correct corresponding points should have a maximum of the correlation value. In this work, a commonly used criterion called normalized cross-correlation (NCC) is used. Suppose $S_{1}$ and $S_{2}$ are two pixel subsets, and the NCC between them is given by:

(4) $$C_{NCC}\left(S_{1},S_{2}\right)=\sum_{u,v}\frac{S_{1}\left(u,v\right)\times S_{2}\left(u^{\prime },v^{\prime }\right)}{\sqrt{\sum S_{1}{\left(u,v\right)}^{2}}\sqrt{{\sum S_{2}\left(u^{\prime },v^{\prime }\right)}^{2}}}.$$

Therefore, the phase ambiguity is eliminated and the accurate corresponding points can be obtained through the proposed comprehensive constraint.

Based on the above techniques, only three phase-shifting images are required to achieve 3D measurement and the efficiency can be remarkably improved. The ultrafast 3D topography monitoring of the PBF AM process is realized. It is capable of monitoring the geometric signatures including flatness, homogeneity and some defects of the powder bed. At the same time, warping, non-uniformity and other defects of fusion area can be also detected by this method. Some experimental results are shown in Section 2.

### 2.3. Rapid and Accurate Contour Detection Assisted by the Slice Model

Contour detection is a fundamental task in image processing and computer vision that has been dealt with from many different approaches [26]. For the proposed in situ 3D monitoring system for process signatures, accurate contour detection is one of the most time-consuming procedures for realizing the 3D contour reconstruction. At the same time, the detection precision dominates the measurement precision once the system mechanism has been determined. In this application, a very high precision in parameters of the contours is required. Therefore, the level set methods [30] is utilized for the sub-pixel precision and outstanding noise robustness performance. The level set methods are a conceptual framework for using level sets as a tool for numerical analysis of surfaces and shapes. In image processing and computer vision applications, the level set methods have been widely used for image segmentation and active contour detection [23,24,25,31]. Let Ω be the image domain of a captured image *I*. A segmentation of the image *I* is achieved by finding the contour *C*. This can be formulated as a problem of minimizing the following Mumford–Shah functional:

(5) $$F^{MS}\left(u,C\right)=\int_{\Omega }{\left(I-u\right)}^{2}dx+\int_{\Omega /C}{\left|\nabla u\right|}^{2}dx+\nu \left|C\right|,$$

where *u* is the piecewise smooth function that approximates the image *I* inside Ω, $\int_{\Omega }{\left(I-u\right)}^{2}dx$ is the data term that forces *u* to be close to image *I*, $\int_{\Omega /C}{\left|\nabla u\right|}^{2}dx$ is the smoothing term that forces *u* to be smooth inside the contour *C*, and the last term is introduced to regularize the contour. As the basic principles discussed above [23,24,25], although the level set methods are of high precision, they are computationally expensive and sensitive to the initialization of the contour. The most time-consuming part when using the level set methods is the huge numbers of iterations without an initial set-up for the contour area. At the same time, some un-wanted false contours out of the fusion area can be also detected. Hence, manual interventions are required to further select the interested data. The above un-acceptable conditions greatly reduce the efficiency and robustness of the contour detection and reconstruction procedure. In order to accelerate the calculation time and increase the accuracy of the level set method, some prior knowledge given by the slice model can be utilized to assist the process.

**Figure 3:**
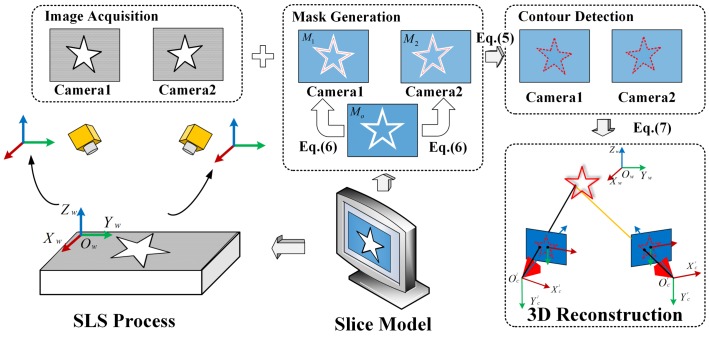
Procedures to detect the contour position aided by a slice model.

The procedure to detect the contour position and to do 3D reconstruction are summarized in the following steps, as schematically shown in Figure 3. First, the laser sinters the powder according to a pre-defined scanning path generated by the slice model. A series of surface images of the fusion area, which are captured by the left and right cameras, are recorded using the established stereo vision system once the layer is scanned by the laser. Subsequently, an original mask $M_{o}$ is generated from the slice model by some image erosion operations. As shown in Figure 3, the relationships between the manufacture coordinate system and camera coordinate systems ($R_{1},T_{1}$ and $R_{2},T_{2}$ ) can be pre-calibrated by the Perspective-N-Point method [29]. The original mask $M_{o}$ is then processed to create the appropriate image masks for camera 1 and camera 2, respectively:

(6) $$\left\{\begin{array}{c} M_{1}=A_{1}\left[\begin{array}{cc} R_{1} & T_{1} \end{array}\right]M_{o},\\ M_{2}=A_{2}\left[\begin{array}{cc} R_{2} & T_{2} \end{array}\right]M_{o}, \end{array}\right.$$

where $A_{1}$ and $A_{2}$ are the intrinsic parameters of cameras 1 and 2, and $M_{1}$ and $M_{2}$ are the calculated image masks. The image masks are utilized to generate the Region of Interest (ROI)s $\Omega_{M_{1}}$ and $\Omega_{M_{2}}$ on the recorded images. Then, the level sets method is conducted to detect the contours just inside the ROIs $\Omega_{M_{1}}$ and $\Omega_{M_{2}}$ to avoid extraction failures and noises. Suppose one detected contour point in the left camera is $p_{1}$ and the corresponding point is $p_{2}$ in the right camera. One can reconstruct the 3D contour data $P_{w}$ by a least-squares solution according to the following equation:

(7) $$\left\{\begin{array}{c} s_{1}{\tilde{p}}_{1}=A_{1}\left[R_{1}|T_{1}\right]{\tilde{P}}_{w},\\ s_{2}{\tilde{p}}_{2}=A_{2}\left[R_{2}|T_{2}\right]{\tilde{P}}_{w}, \end{array}\right.$$

where ${\tilde{p}}_{1},{\tilde{p}}_{2},{\tilde{P}}_{w}$ are the homogenous coordinates of $p_{1},p_{2},P_{w}$, respectively, by adding 1 after the last element. $s_{1},s_{2}$ are scale factors.$A_{1},A_{2},R_{1},T_{1},R_{2},T_{2}$ can be accurately calibrated before measurements. The proposed method significantly increases the effectiveness and accuracy of the contour detection process significantly. Based on the binocular stereoscopic vision algorithms, the 3D contour data can be calculated. The experimental results are shown in the following section.

## 3. Experiments

**Figure 4:**
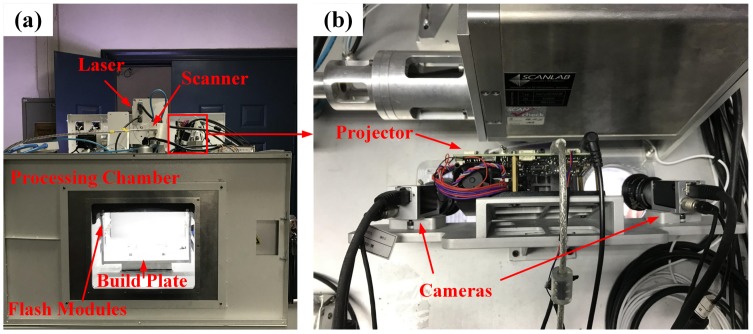
Experimental arrangements for in situ geometric process signatures metrology of the PBF AM process.

To verify the accuracy and effectiveness of the established in situ monitoring method for the geometric process signatures metrology of the PBF AM process, experiments were conducted including the measurement of the powder bed, fusion area, and 3D contour data. Figure 4 shows the experimental arrangements. The in situ monitoring system utilized two industrial Charge-coupled Device (CCD) cameras (Basler ace-acA2500-14gm) with the image size up to 2592×1944 pixels. The projector was composed of a Texas Instruments LighterCrafter 4500 board with a 150 ANSI lumens RGB-LED. The cameras and projector were synchronized by an external triggering circuit at a frame rate of 30 Fps. The sintering experiments were conducted on the HK S320TM SLS machine (Wuhan Huake 3D Technology Co. Ltd, Wuhan, China). The SLS system was equipped with a power continuously adjustable $CO_{2}$ laser with a wavelength and beam diameter of 10.6 μm and 200 μm, respectively.

### 3.1. 3D Surface Topography Monitoring of the Powder Bed

**Figure 5:**
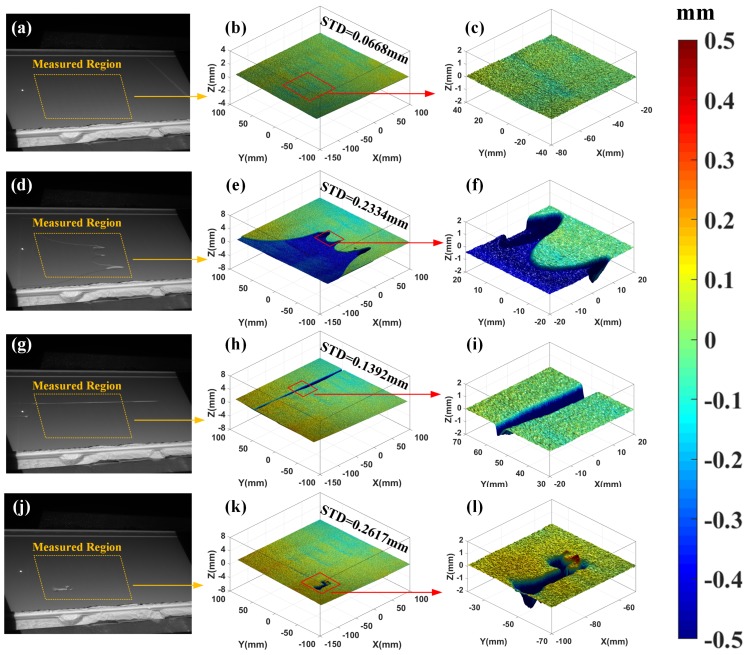
Measurement results of the powder bed topography using the proposed Enhanced PMP framework. The first row shows the normal condition after powder composing: (**a**) the real scene, (**b**) integral 3D topography, (**c**) local topography of selected region; The second row shows the flawed condition of lacking powder: (**d**) the real scene, (**e**) integral 3D topography, (**f**) local topography of the selected region; The third row shows the flawed condition due to the recoater: (**g**) the real scene, (**h**) integral 3D topography, (**i**) local topography of the selected region; The fourth row shows the flawed condition due to small holes: (**j**) the real scene, (**k**) integral 3D topography, (**l**) local topography of the selected region.

By processing the recorded raster images of the powder bed after powder depositing, 3D full-field surface data can be retrieved. Figure 5 shows the defects of the powder depositing and the directly measured 3D data of the powder bed, respectively. As clearly presented in these figures, the 3D height maps of defects hold different standard deviation values. Figure 5a–c show the normal condition after the new layer of powder is spread on the powder bed. The height map provides rich information about the powder depositing process. The powder bed is not in an ideal plane and there are some small stripes on the whole powder bed plane. These indistinct defects can be hardly extracted from 2D digital images of the surface and they may contribute to improving the mechanism of the powder depositing process. Three different defects are characterized by the proposed method as shown in Figure 5d–l including lack of powder, defects of the recoater and holes of the powder bed. The second row shows the flawed condition of lacking powder. The standard deviation (Std) of the 3D height map is 0.2334 mm, which is about four times that of the normal condition. The area and position of the flawed region can be also calculated from the measured data quantitatively. The third row presents the powder spreading defects due to the recoater. It usually results in unexpected grooves in the powder bed surface. The measurement results can present the depth and width of the grooves as shown in Figure 5i. The standard deviation (Std) is about two times that of the normal condition. The fourth row shows the flawed condition due to small holes. The holes can be clearly represented in the 3D height maps. According to the above results, once the defects occur, the standard deviations (Std) of the 3D height maps are approximately 2–6 times those of normal conditions. Therefore, the value of Std can be regarded as an important geometric signature of the powder bed. One can also calculate the position, size, and level of the defect region from the measured 3D height maps directly. By utilizing the proposed measurement method, various levels of the geometric signatures of the powder bed can be detected.

### 3.2. 3D Surface Topography Monitoring of the Fusion Area

**Figure 6:**
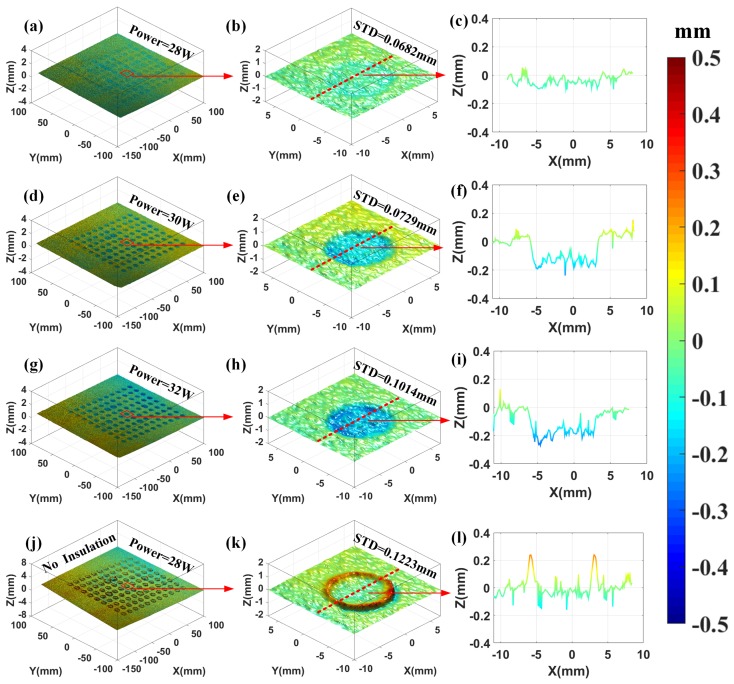
Measurement results of the fusion area topography using the proposed EPMP framework with laser power of 28 W, 30 W, 32 W and 28 W without insulation, respectively. The first row shows the height map of the surface fused with laser power of 28 W: (**a**) integral 3D topography, (**b**) local topography of selected region, (**c**) line profile of the surface along the red line; The second row shows the height map of the surface fused with laser power of 30 W: (**d**) integral 3D topography, (**e**) local topography of selected region, (**f**) line profile of the surface along the red line; The third row shows the height map of the surface fused with laser power of 32 W: (**g**) integral 3D topography, (**h**) local topography of selected region, (**i**) line profile of the surface along the red line; The fourth row shows the height map of the surface fused with laser power of 28 W without insulation: (**j**) integral 3D topography, (**k**) local topography of selected region, (**l**) line profile of the surface along the red line.

Figure 6 shows the in situ height maps of layers fused with different laser powers of 28 W, 30 W, 32 W and 28 W without insulation, respectively. The laser power of 28 W is the optimal parameter for the experimental configuration. Figure 6a–c show the optimal condition with the laser power of 28 W. The selected area has a height variation less than 0.1 mm over the fused and unfused regions with a small Std value of 0.0682 mm. Once we raised the laser power, the height variations and Std value all increased. As shown in the second and third row of Figure 6, the value of height variations and Std are about 0.3 mm, 0.0729 mm and 0.4 mm, 0.1014 mm, respectively. These noticeable signatures can be all well measured or derived by the proposed method quantitatively. Furthermore, one can obtain rich information about the fusion process. Firstly, the average height of the fusion area is lower than that of the unfused powder surface. The sinking phenomenon is primarily due to the solidification process of the powder. Secondly, the amount of the surface sinking increases as the laser power increases and this is consistent with the literature [21]. Thirdly, one can calculate the surface roughness of the fusion area and the boundary of the fused area can be easily defined by the grooves between the fused and unfused regions. To demonstrate the ability to observe defects during the fusion process, a typical defect, warping is produced artificially by decreasing the temperature in the chamber (closing the insulation module) as shown in Figure 6j–l. One can clearly observe the warping phenomenon from the whole 3D height map. Furthermore, the selected warping area holds over 0.3 mm in height variation and a line profile of the selected region is obtained along the red line. From the line profile, the quantitative characterization of the warping can be easily derived by calculating the peak-valley value of the profile as shown in Figure 6l.

### 3.3. 3D Contour Monitoring of the Fusion Area

**Figure 7:**
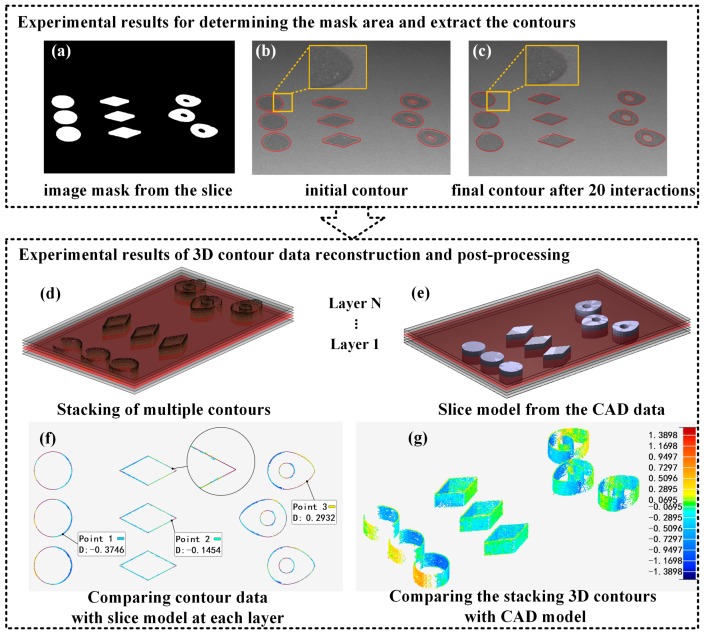
Experimental results of extracting the contours in subpixel precision and 3D contour data reconstruction. (**a**) the generated original mask from the slice, (**b**) the initial contour for the level set method, (**c**) the final contour after 20 interactions, (**d**) layer-wise 3D contour data, (**e**) ground-truth contour data from the slice model, (**f**) contour accuracy at a layer, (**g**) digital 3D volume comparing to the CAD model.

As shown in Figure 7a–c, the proposed contour detection results are given including: (a) the translated image mask of left camera; (b) the initial contour for the level set method; and (c) the final contour after 20 interactions. With the aid of the slice model, the image mask generates a suitable region of interest in the image to be processed at first. This will contribute to improving the accuracy and effectiveness of the level sets method. With a good initialization as shown in Figure 7b, the method can quickly converge to the actual contours in subpixel precision as shown in Figure 7c. The number of iterations is 10–20 in our experiments, which is less than a tenth of the traditional method without slice model assistance.

Then, an unambiguous reconstruction method is conducted to obtain the 3D contour data in this layer as shown in Figure 7d–g. Layer-wise 3D contour data are collected during the fabrication of parts modeled with intentional defects as shown in Figure 7d. For each layer, one can obtain the ground-truth contour data from the slice model as illustrated in Figure 7e. Based on the collected data above, the contour accuracy can be calculated as shown in Figure 7f and it is an important geometric signature for the PBF AM process. According to the contour accuracy results, one can come to a conclusion that the manufacturing accuracy in the platform center is higher than that in the edges. It is probably because of the distortions of the laser galvanom scanning system. With in situ and real-time 3D measurement, it holds the potential to re-calibrate laser the galvanom scanning system in the building process. By stacking all the contour data, a digital 3D volume for the contours, indexed to the part geometry, is created. This offers an opportunity to inspect the outside contours of the solid part in real time, without taking it out from the powder bed. This digital 3D volume is compared to the ground truth data, which is established from the CAD model as illustrated in Figure 7g. Comparing the Figure 7f,g, one can find that the trend of unexpected deformation is similar and the manufacturing precision in the platform center is better.

## 4. Discussion

Compared to existing in situ geometric signatures monitoring methods, our method has the following advantages:

- Unlike traditional phase measuring profilometry (PMP) methods that require additional images (6–12) for phase unwrapping procedure, the enhanced phase measuring profilometry (EPMP) is proposed to measure the 3D surface topography reliably without phase unwrapping. The efficiency can be remarkably improved, which means, in this case, the demands concerning measurement accuracy and processing velocity.
- The 3D contour data of each layer of fusion area is measured first. The slice model assisted contour detection method is proposed to increase the accuracy and efficiency of the contour detection process. The manual interventions are no longer needed and the contour detection process can operate automatically.
- The measured geometric signatures can contribute to adjusting the process parameters for the PBF AM process. It also offers a viable tool for manufacturing process visualization.

However, the proposed in situ 3D monitoring method still has room for further improvement.

- *Real-time close-loop control*. According to a roadmap workshop on the measurement science needs for metal-based AM [7], real-time close-loop control systems for AM was identified as an important technology. The proposed method is a kind of 3D sensing technique that can monitor two categories of geometric signatures including 3D surface topography properties and 3D contour data. Therefore, it can be regarded as the “eyes” of control system and it is vital for realizing the real-time close-loop control systems for AM. Our future work will focus on building the close-loop control strategies based on the proposed 3D monitoring techniques and gaining the ability to qualify and certify parts and processes.
- *Intelligent recognition and classification of defects*. On the one hand, the stated AM system is equipped with proposed in situ sensing devices able to measure relevant quantities during the process, a.k.a. height profile, homogeneity and flatness of the powder layer, and surface irregularities of the fused regions. On the other hand, in-process data analytics and machine learning techniques are required to detect, localize and identify the defects in an automated way. Our future work will also focus on combining the 3D sensing techniques with some artificial intelligence algorithms to realize intelligent recognition and classification of defects.

## 5. Conclusions

This paper presents an in situ 3D monitoring method of geometric process signatures in PBF AM process via vision sensing methods. The first category of geometric process signatures is 3D surface topography properties. To increase the measurement efficiency and accuracy, an enhanced phase measuring profilometry (EPMP) is proposed to monitor the 3D surface topography reliably under the comprehensive constraint. It can monitor the geometric signatures including the height profile, the homogeneity and flatness of the powder layer, and surface irregularities of the fused regions, etc. The analysis of the 3D surface topography properties can reveal irregularities caused by different defects. Another important category of geometric process signatures concerned in this work is the 3D contour data of the fusion area. A rapid and accurate contour detection assisted by slice model is developed based on the level set methods. The proposed method increases the effectiveness and accuracy of the contour detection process significantly. The measurement of contour data allows implementation of a metrological system able to reconstruct the actual 3D shape of the printed slice on a layer-by-layer basis. The contour accuracy at each layer and the overall accuracy can all be derived from the measurement data. The in situ 3D monitoring method for geometric signatures outlined will contribute to the development of addictive manufacturing on many levels, such as inspecting the contour accuracy and surface quality. It also holds the potential to be a powerful tool for manufacturing process optimization, close-loop control, and data visualization.
